# Comparison of Keratometric Change Following Various Conjunctival Autografting Techniques in Pterygium Surgery

**DOI:** 10.7759/cureus.49662

**Published:** 2023-11-29

**Authors:** Snigdha Sen, Anu Jain, Prerna Upadhyay, Merensoba T Imchen, Tirupati Nath, Akash Kakkar

**Affiliations:** 1 Department of Ophthalmology, Sarojini Naidu Medical College, Agra, IND; 2 Department of Ophthalmology, Government Medical College, Autonomous State Medical College, Firozabad, IND

**Keywords:** autologous blood, fibrin glue, conjunctival auto grafting, pterygium surgery, pterygium

## Abstract

Aim

To compare the postoperative keratometric changes and duration of surgery for different techniques of conjunctival autografting in pterygium surgery.

Methods

Patients with primary pterygium attending the outpatient department and having appropriate indications for surgery were enrolled. Preoperative ophthalmic examination included visual acuity assessment, refraction, keratometry, slit lamp, and fundus evaluation. Pterygium excision surgery with conjunctival autografting was performed on all patients using one of the four different techniques, namely, sutures, fibrin glue, and the autologous blood and bridge techniques. Duration of surgery was recorded for all patients. Postoperatively, all patients were followed up on Day 1, Day 7, and Months 1, 3, and 6. Duration of surgery, keratometric changes, and recurrence rates were analyzed in all four groups.

Results

Sixty-eight eyes of 66 patients completed the study protocol. There was a significant reduction in astigmatism after the autologous blood graft technique (*P* value 0.0055) and the glue technique (*P* value < 0.0001). The success rate of the autologous and glue technique was 90%. The glue technique was found to be more time efficient (mean duration 20.40 minutes) than other techniques.

Conclusion

After pterygium excision, conjunctival auto grafting using either autologous blood or glue plays a significant role in reducing pterygium-induced astigmatism and recurrence rates with the added advantage of a shorter operative time.

## Introduction

The pterygium is wedge-shaped fibro-vascular dysplasia of the bulbar conjunctiva and is usually located on the nasal, or rarely, on the temporal side. Though it occurs worldwide, its prevalence is higher in the pterygium belt between 40 degrees north and 40 degrees south of the equator due to the higher intensity of ultraviolet radiation in the region [1]. The prevalence of pterygium has been variously reported as about 3-30% from different parts of the globe [2].

Pterygium leads to visual impairment by invading the visual axis as well as by distorting the corneal topography and inducing astigmatism. Apart from visual impairment, pterygium excision is also indicated due to recurrent inflammation, motility disturbance, and cosmetic disfigurement. Conjunctival autografting plays a key role in the surgical management of pterygium, thereby reducing the recurrence rate, which is otherwise a major concern following the bare sclera technique of pterygium excision [3]. The graft can be repositioned on the sclera with either sutures, fibrin glue, or autologous blood.

We intended to compare the operative time and keratometric change of various conjunctival auto-grafting techniques, namely, with suture, fibrin glue, and autologous blood along with the bridge technique of conjunctival flapping.

## Materials and methods

After obtaining ethical clearance from the Institutional Ethics Committee, a prospective randomized observational study was undertaken at our tertiary care center on patients aged >18 years who presented with primary nasal pterygium. Exclusion criteria included atrophic pterygium, patients who were taking anticoagulants; immune systemic diseases, such as lupus or rheumatoid arthritis; eyelid or ocular surface diseases, such as blepharitis, Sjogren syndrome, or dry eye; and history of previous ocular surgery or trauma, which may have altered the anatomy or function of the eye, thus complicating the planned surgical procedure.

Written informed consent for pterygium surgery was obtained from all patients after explaining to them the expected favorable change in postoperative astigmatism and vision as well as the chances of recurrence at a later date.

Preoperative evaluation included visual acuity assessment, refraction, keratometry (Bausch and Lomb Keratometer; Rochester, New York), slit lamp, and fundus examination. Grading of the pterygium was done according to the amount of corneal invasion, measured on a slit lamp, from limbus up to the edge of the pterygium head. Corneal invasion of <2 millimeters (mm) was categorized as grade 1, that of 2-4 mm as grade 2, and that of >4 mm as grade 3.

Horizontal and vertical keratometric values were measured precisely preoperatively as well as postoperatively and at each follow-up visit. Each measurement was repeated at least three times until the discrepancy of keratometric data was less than 0.25 diopters (D). Because the principal axes of all the patients were not along perfect horizontal (180-degree) and vertical (90-degree) meridia, the horizontal K was taken as the K reading at the principal axis nearer to the 180-degree meridian and the vertical K, as the K reading near the 90-degree meridian. Due to the unavailability of a corneal topography machine and an optical biometer, software-based corneal topographic parameters could not be assessed.

Prior to the surgical procedure, the patients were randomly assigned following simple randomization procedures (using computer-generated random numbers) to one of the four surgical groups as follows: Group (A) - Autologous Blood Technique, Group (B) - Suture Technique, Group (C) - Fibrin Glue Technique, and Group (D) - Bridge Technique.

**Figure 1:**
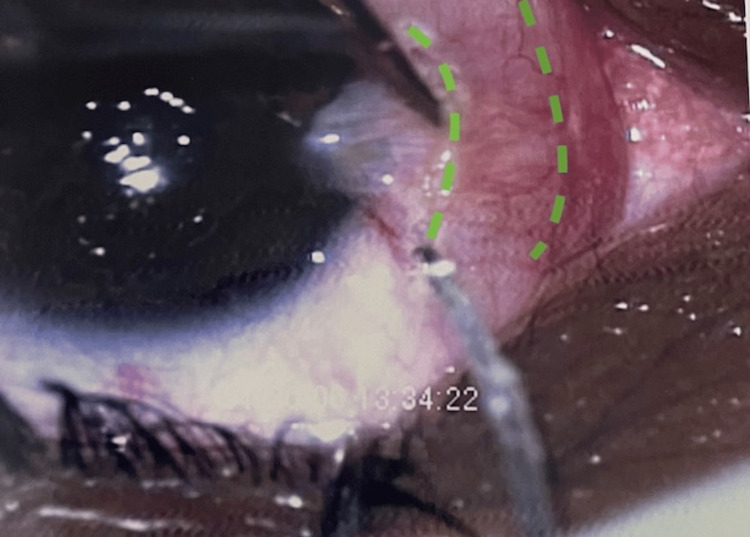
Placement of parallel incisions (green dotted lines) on the conjunctiva overlying the pterygium in the bridge technique of pterygium excision and conjunctival flapping

The surgical procedure was carried out by a single surgeon (SS) under peribulbar anesthesia and involved excising the pterygium tissue and a 1 mm margin of the conjunctiva, creating a space under the healthy conjunctiva around the defect, and measuring the size of the bare sclera. A conjunctival graft was taken from the superotemporal area, dissected off the underlying tenon, and cut from the limbus. The graft was repositioned on the bare sclera with the limbal edge of the graft aligned with the limbus. In Group (A) patients, the blood under the graft was allowed to clot for 3-4 minutes. In Group (B) patients, the autograft was sutured to the adjacent conjunctiva and to the underlying sclera at the limbus using 10-0 nylon interrupted sutures. For patients in the suture group, the protocol was to remove the sutures after four weeks. However, during the routine follow-ups, if any of the patients had discomfort, they were removed but not earlier than two weeks. For Group (C) patients, the fibrin sealant vial (blue label) and the thrombin sealant vial (black label) were used to prepare two syringes with 0.1 cc of fibrinogen and 0.1 cc of thrombin mixed with 0.9 cc of balanced salt solution each. A drop of fibrinogen was placed on the recipient site followed by the placement of graft on the site after which a drop of thrombin was instilled over the graft. The graft was left undisturbed for 30 seconds before patching the eye. Group (D) patients underwent pterygium excision employing the bridge technique. In the bridge technique, two vertical parallel incisions were made in the conjunctiva over the pterygium, one close to the limbus at the neck of the pterygium and another 1-2 mm lateral to the canthal end, fashioning a bridge of conjunctiva overlying the pterygium (Figure 1). The pterygium mass was meticulously dissected subconjunctivally and separated from the underlying episclera and the cornea. The dissected pterygium mass was excised, and the conjunctival flap remained in place covering the sclera. The remaining corneal part of the pterygium was scraped with the help of a scalpel. At the end of the surgical procedure, the eye was patched with an antibiotic-steroid ointment.

In order to decimate any inter-observer bias, preoperative workup and postoperative/follow-up evaluation in all the cases was performed by a single ophthalmologist (AK), other than the operating surgeon (SS). Duration of surgery was recorded for each patient. The post-surgery treatment protocol was common in all four study groups and comprised moxifloxacin (0.5%) - dexamethasone (0.1%) eyedrops in tapering doses over one month and carboxy methyl cellulose (1%) eyedrops.

Postoperative evaluation was carried out on Day 1, Day 7, and at Months 1, 3, and 6 thereafter. Day 1 and Day 7 follow-ups were done to look for early postop complications such as edema and graft displacement. The cases, where graft displacement was encountered during the initial postoperative period and required reinforcement with sutures, were excluded from further evaluation. The first, third, and sixth-month follow-ups recorded the changes in corneal contour and improvements, if any, of the astigmatism induced by the condition. Recurrence was defined as any regrowth of fibrovascular tissue over the cornea.

Statistical analysis was performed using SPSS for Windows (Version 16.0, 2007; SPSS Inc., Chicago, IL, USA). Paired t-test was used to compare the variables. The probability level of 0.05 was set as the statistically significant value.

## Results

Sixty-eight eyes of 66 patients with a mean age of 48.7 + 10.98 years and a male-female ratio of about 2.1:1 completed the study protocol. The most common presenting complaint was the presence of abnormal growth (48.88%), followed by redness and watering. The other symptoms, depending upon the extent of corneal involvement and thickness of the pterygium, included restricted ocular movement and decreased vision.

Three out of 21 eyes (14.28%) in the autologous blood group and 2 of 18 eyes (11.11%) in the fibrin glue group showed graft displacement during the early postoperative period and required reinforcement with sutures. These cases were omitted from being part of further evaluation. Table 1 depicts the characteristics and mean preoperative keratometry readings of patients allocated to different surgery groups. Almost about half of the patients had pterygium grade 2 (corneal invasion between 2 and 4 mm).

**Table 1:** Patient characteristics and preoperative keratometric values *KH (Horizontal Keratometry Value); KV (Vertical Keratometry Value)

		Group A	Group B	Group C	Group D	Total
No. of Eyes (n)		18	17	16	17	68
Mean Age, Years (SD)		51. 5 (9.02)	48.4 (10.72)	49.6 (8.98)	51.9 (7.71)	48.7 (10.98)
Gender	M	12	10	11	13	46
	F	6	7	5	4	22
Grade of Pterygium	1	5	4	6	5	20
	2	8	8	8	8	32
	3	5	5	2	4	16
Mean Pre-op Keratometry^*^ (Diopters)	KH	41.33	42.85	43.20	43.93	42.83
	KV	42.50	43.60	44.20	44.60	43.72

The mean operative duration was recorded as the maximum for the bridge technique group. Pterygium surgery with conjunctival autografting using fibrin glue turned out to be the most time-efficient technique (Table 2).

**Table 2:** Mean operative duration in different study groups

Study Group	Mean Operative Time (S.D.), minutes
A (Autologous blood)	35.10 (7.97)
B (Suture)	41.83 (4.56)
C (Fibrin glue)	20.40 (2.87)
D (Bridge technique)	50.70 (4.01)

Table 3 compares the preoperative keratometry readings with postoperative keratometric power in different groups. A significant difference was found in horizontal keratometric value in group A (p-value-0.0055) and group C (p-value <0.0001) at the end of three months whereas changes in keratometric values in the rest of the groups were statistically insignificant. At the sixth-month follow-up, the change in keratometric values was similar to that at three months across all the study groups. This implied that by three months post-pterygium surgery, most of the patients had attained corneal surface refractive stability.

**Table 3:** Comparison of pre- and postoperative keratometry value (in Diopter) at the end of three months *KH (Horizontal Keratometry Value); KV (Vertical Keratometry Value) ^# ^The paired t-test was used to derive the p-value.

Group	Mean Preop Keratometric Value		Mean 3-Month Keratometric Value		% Change		p-value^#^	
	KH	KV	KH	KV	KH	KV	KH	KV
Group A	41.33	42.50	42.75	42.93	3.44	1.01	0.0055	0.4051
Group B	42.85	43.60	43.38	43.55	1.24	-0.11	0.6289	0.9461
Group C	43.20	44.20	44.10	44.23	3.79	2.38	<0.0001	0.0107
Group D	43.93	44.60	44.33	44.58	0.91	-0.04	0.5107	0.9803

Table 4 shows that Group A and Group C had the greatest reduction in KV-KH values in the postoperative follow-up with respect to their values in the preoperative assessment with the percentage of reduction being 84.62% for Group A and with a slightly higher reduction for Group C at 87.00%.

**Table 4:** Comparison of mean pre- and postoperative KV-KH *KH (Horizontal Keratometry Value); KV (Vertical Keratometry Value)

GROUP	Pre-Op			Postop (3 months)			Mean difference in preop and postop KV-KH	Percentage reduction between preop and postop KV-KH
	Mean KH	Mean KV	Mean KV-KH	Mean KH	Mean KV	Mean KV- KH		
A	41.33	42.5	1.17	42.75	42.93	0.18	0.99	84.62
B	42.85	43.6	0.75	43.38	43.55	0.17	0.58	77.33
C	43.2	44.2	1	44.1	44.23	0.13	0.87	87.00
D	43.93	44.6	0.67	44.33	44.58	0.25	0.42	62.69

The overall recurrence rate at the six-month follow-up was found to be 10.29%. Most eyes that showed recurrence were in the group that underwent the bridge technique, followed by those in the suture group (Table 5).

**Table 5:** Number of eyes with recurrence at the six-month follow-up

Study group	n	No. of eyes with recurrence	Percentage (%)
A	18	1	5.56
B	17	2	11.76
C	16	1	6.25
D	17	3	17.64
Total	68	7	10.29

## Discussion

Pterygium is a common conjunctival disorder with a relatively unambiguous diagnosis but challenging management. Multiple surgical techniques and modifications have been developed to manage the condition and prevent recurrence. Traditional pterygium excision with bare sclera has fallen into disrepute due to the high recurrence rate and potential complications such as scleral thinning, necrotizing scleritis, infection, and delayed wound healing [4,5]. Pterygium excision with conjunctival autografting significantly reduces recurrence rates and leads to fewer complications, and, therefore, has become the surgical procedure of choice [4,6]. Traditionally, sutures have been used to attach the graft to the underlying sclera. Recently, the use of fibrin glue and blood coagulum to secure the graft, instead of sutures has become popular. The use of autologous cryo-precipitate has also been described for attaching the graft [7].

As has been reported earlier, the pterygium results in high corneal astigmatism, which decreases following an excision [8,10]. Pterygium size is the most important determinant in postoperative astigmatic change [8,11]. Errais et al. found a significant change in keratometric value after conjunctival autografting with the suture technique [12]. The present study intended to evaluate if different conjunctival autografting techniques differed in eliciting desired change in postoperative astigmatism. We observed that the postoperative astigmatism decreased significantly in the autologous blood group and the fibrin glue group at the end of three months while the astigmatic change was insignificant in the other two study groups.

Altan-Yaycioglu and co-workers found that the type of grafting as autograft, rotational flap, or amniotic membrane or the use of suture or glue to fixate the graft does not have a statistically significant effect on the change in astigmatism [13]. Wang observed no statistically significant difference in visual acuity gain following conjunctival autografting using sutures or fibrin glue [14]. Another comparative study from the Indian subcontinent concluded that amniotic membrane graft and conjunctival autograft are better surgical techniques than bare sclera as far as reducing astigmatism is concerned [9]. So although most of the previous studies demonstrated equivocal outcomes following different surgical techniques, in terms of postoperative astigmatic change, our study observed significant astigmatic change with fibrin glue and autologous blood use for conjunctival grafting. Authors, thus, hypothesize that fibrin glue or autologous blood being a more bio-compatible option as compared to sutures, results in less granulation tissue formation and better healing, when used for conjunctival grafting [15].

The early postoperative complications of pterygium excision followed by conjunctival autografting include subconjunctival hemorrhage, graft edema, graft loss, retraction, graft displacement, and cyst/granuloma formation [16]. In our study, graft dislodgement in the early postoperative period was seen in 14.28% and 11.11% cases of the autologous blood group and the fibrin glue group, respectively, requiring re-enforcement with sutures. No other graft-related complications were observed.

The overall recurrence rate at the six-month follow-up was observed to be 10.29%, with the least and comparable recurrence rate among the fibrin glue and autologous blood groups and the highest among the bridge technique patients. The recurrence rate was noted as 11.76% among patients who underwent graft suturing. The recurrence rate following conjunctival autografting, irrespective of the technique, has been previously reported to vary from 2-39% [17]. In a recent meta-analysis, the recurrence rate at six months was reported as 3.33-16.7% in the conjunctival autograft groups and 2.6-42.3% in the amniotic membrane transplant groups [3]. Farid and coworkers, in a retrospective study of 47 eyes, found that the recurrence rate in the tissue adhesive group was 3.7% compared to 20% in the suture group, which is quite higher than that in our study [18]. It has been postulated that gluing the conjunctiva inhibits fibroblast migration into the surgical area, thus minimizing the risk of recurrence. On the other hand, few other studies have observed no statistically significant difference in the recurrence rates between the suture and fibrin glue group at the end of the sixth-month postoperative follow-up period [14,19,20].

In this study, the mean operative time for study groups using the glue technique and the suture technique was 20.40 minutes and 41.83 minutes, respectively. This was comparable to another study by Karalezli et al. in which the mean surgical time in the fibrin glue group was 15.7 minutes and in the suture group was 32.5 minutes [21]. Few other studies compared the duration of surgery with fibrin glue and suturing among patients undergoing pterygium excision and found that the average operating time for the fibrin glue group was significantly lesser [17,22]. In another study that included the use of autologous blood in one of the study groups, the average surgical time was the least with the fibrin glue group (36.2 mins), followed by that of the autologous blood group (44.8 min) and maximum (53.3 min) with the suture group [23]. The bridge technique, in the present study, was found to be the most time-consuming procedure with a mean duration of 50.70 minutes.

The present study is one of the very few studies that assessed the corneal astigmatic change following different techniques of conjunctival autografting. However, the results of the study would have been more categorical with the use of a corneal topography machine and an optical biometer, instead of a keratometer alone.

## Conclusions

Pterygium‑induced astigmatism can be significantly reduced by surgical excision, thereby resulting in an improvement in visual acuity. There was a significant reduction in corneal astigmatism in both the autologous blood and fibrin glue groups. However, we recommend the use of fibrin glue as the preferred option for conjunctival auto-grafting in terms of less graft displacement, better patient tolerance, the least recurrence rate, and most time-efficient surgical technique.
